# Effects of mindful-attention and compassion meditation training on amygdala response to emotional stimuli in an ordinary, non-meditative state

**DOI:** 10.3389/fnhum.2012.00292

**Published:** 2012-11-01

**Authors:** Gaëlle Desbordes, Lobsang T. Negi, Thaddeus W. W. Pace, B. Alan Wallace, Charles L. Raison, Eric L. Schwartz

**Affiliations:** ^1^Athinoula A. Martinos Center for Biomedical Imaging, Massachusetts General HospitalBoston, MA, USA; ^2^Center for Computational Neuroscience and Neural Technology, Boston UniversityBoston, MA, USA; ^3^Department of Religion, Emory UniversityAtlanta, GA, USA; ^4^Department of Psychiatry and Behavioral Sciences, Emory University School of MedicineAtlanta, GA, USA; ^5^Santa Barbara Institute for Consciousness StudiesSanta Barbara, CA, USA; ^6^Department of Psychiatry, College of Medicine and Norton School of Family and Consumer Sciences, College of Agriculture, University of ArizonaTucson, AZ, USA; ^7^Department of Electrical and Computer Engineering, Boston UniversityBoston, MA, USA

**Keywords:** meditation, mindfulness, attention, compassion, amygdala, emotion, fMRI

## Abstract

The amygdala has been repeatedly implicated in emotional processing of both positive and negative-valence stimuli. Previous studies suggest that the amygdala response to emotional stimuli is lower when the subject is in a meditative state of mindful-attention, both in beginner meditators after an 8-week meditation intervention and in expert meditators. However, the longitudinal effects of meditation training on amygdala responses have not been reported when participants are in an ordinary, non-meditative state. In this study, we investigated how 8 weeks of training in meditation affects amygdala responses to emotional stimuli in subjects when in a non-meditative state. Healthy adults with no prior meditation experience took part in 8 weeks of either Mindful Attention Training (MAT), Cognitively-Based Compassion Training (CBCT; a program based on Tibetan Buddhist compassion meditation practices), or an active control intervention. Before and after the intervention, participants underwent an fMRI experiment during which they were presented images with positive, negative, and neutral emotional valences from the IAPS database while remaining in an ordinary, non-meditative state. Using a region-of-interest analysis, we found a longitudinal decrease in right amygdala activation in the Mindful Attention group in response to positive images, and in response to images of all valences overall. In the CBCT group, we found a trend increase in right amygdala response to negative images, which was significantly correlated with a decrease in depression score. No effects or trends were observed in the control group. This finding suggests that the effects of meditation training on emotional processing might transfer to non-meditative states. This is consistent with the hypothesis that meditation training may induce learning that is not stimulus- or task-specific, but process-specific, and thereby may result in enduring changes in mental function.

## Introduction

Meditative practices have generated much interest in the scientific community, in particular with regards to how meditation affects brain function (Lutz et al., 2007; Austin, 2009; Slagter et al., 2011). While meditative states are interesting to study *per se*, perhaps more intriguing is the possibility that meditation training leads to enduring changes in brain function, even outside meditation sessions (Slagter et al., 2011).

Contemplative practices purportedly lead to increased well-being (e.g., Dalai Lama and Cutler, 1998), a claim supported by subjective reports of participants in mindfulness-based interventions (reviewed in Grossman et al., 2004; Chambers et al., 2009; Rubia, 2009). It has been proposed that these beneficial effects of meditation training may be due to improvements in attentional skills, which are themselves associated with better emotion regulation skills (Chambers et al., 2009; Wadlinger and Isaacowitz, 2011). Accumulating evidence suggests that meditation training yields improved emotional regulation, both in clinical and non-clinical populations. In non-clinical populations mindfulness-based interventions have been associated with lowered intensity and frequency of negative affect (Brown and Ryan, 2003; Chambers et al., 2008), reduced anxiety (Shapiro et al., 1998), more adaptive responding to stress (Davidson et al., 2003), decreased ego-defensive responsivity under threat (Brown et al., 2008), decreases in difficulties regulating emotions (Robins et al., 2012), reduced emotional interference from unpleasant stimuli (Ortner et al., 2007), and less prolonged physiological reactivity to emotional stimuli, in the form of decreased autonomic arousal (skin conductance response) (Ortner et al., 2007).

The interactions between attention and emotion regulation are complex, and likely involve several interrelated brain networks. One brain region that is centrally involved in emotional processing and the interactions between attention and emotion is the amygdala (Davis and Whalen, 2001; Phelps, 2006; Pessoa, 2008). The amygdala facilitates attention toward emotionally significant, or relevant, stimuli (Whalen, 1998; Sander et al., 2003; Vuilleumier, 2005; Whalen and Phelps, 2009). It is involved with attending to and encoding emotional stimuli, learning about the emotional significance of potentially ambiguous stimuli, distinguishing threat from safety, and appraising and responding to emotionally significant events—including stimuli of both positive and negative valence (Baxter and Murray, 2002; Phan et al., 2002; Sander et al., 2003; Zald, 2003; Haas and Canli, 2008; Sergerie et al., 2008; reviewed in Whalen and Phelps, 2009). Interestingly, Schaefer et al. (2002) found that amygdala activation could be voluntarily increased when subjects were asked to “maintain” the emotional response to negative-valence stimuli, and the amount of amygdala activation increase was correlated with subjects' self-reported dispositional levels of negative affect. Conversely, decreased amygdala activation was observed during the application of emotional regulation strategies such as cognitive distancing and reappraisal (Beauregard et al., 2001; Ochsner et al., 2002, 2004; Lévesque et al., 2003).

While it is well-known that amygdala function is impaired in a number of disorders including depression, anxiety, and post-traumatic stress disorder, amygdala activation also differs across healthy individuals according to their personality traits (Davidson, 1998; Davidson and Irwin, 1999; Lapate et al., 2012). Individuals differ in how they attend to, process, and remember emotional stimuli. Individual differences in personality traits can be traced to a brain attentional network driven primarily by amygdala reactivity during the encoding of emotional stimuli (Haas and Canli, 2008). For example, Fischer et al. (2001) found that amygdala activation while viewing fear-eliciting stimuli was correlated with dispositional pessimism. Canli and colleagues found that amygdala response to positive and negative-valence stimuli was correlated with the personality traits of extraversion and neuroticism (Canli et al., 2001, 2002).

Individuals also vary in their propensity to engage in spontaneous emotion regulation strategies, such as reappraisal or suppression. Spontaneous reappraisal tendencies have been associated with lower levels of negative affect, greater interpersonal functioning, and greater psychological and physical well-being, and the opposite was found for spontaneous tendencies for emotional suppression (Gross and John, 2003). Individual differences in self-reported reappraisal tendencies were associated with decreased amygdala activity during the processing of negative emotional facial expressions (Drabant et al., 2009), pointing again at a crucial role of the amygdala in trait-like emotion regulation skills.

Given the association between amygdala activation and trait emotion regulation and attention, and given the hypothesis that meditation training may contribute to the development of such traits (Slagter et al., 2011), the question naturally arises as to whether amygdala activation may be modified by meditation training. Previous studies indicate that this might be the case. Several neuroimaging studies have implicated the amygdala in the effects of meditation training on the brain. In participants without prior meditation experience, mindfulness meditation training was associated with lower amygdala response to emotional stimuli when the subject entered a meditative state of mindful-attention, both in patients with social anxiety disorder (Goldin and Gross, 2010) and in healthy subjects (Taylor et al., 2011). Similar results have been reported in highly experienced meditation practitioners (Brefczynski-Lewis et al., 2007; but see Taylor et al., 2011). While the above studies investigated meditative states, it has also been proposed that meditation training may induce learning that is not stimulus- or task-specific, but process-specific, and thereby may result in enduring changes in mental function (Lutz et al., 2007, 2008b). These changes should correspond to changes in the brain that outlast the functional changes measured during meditation, and would be more indicative of a change in trait. Some support for this hypothesis can be found in structural differences in the brain which have been reported in relation to meditation training, both cross-sectionally in comparing experienced meditators with matched, meditation-naïve controls (Lazar et al., 2005; Pagnoni and Cekic, 2007; Luders et al., 2009, 2011; Grant et al., 2010), and longitudinally as measured before and after a meditation-based intervention (Hölzel et al., 2010, 2011a; Tang et al., 2010). In addition, several recent cross-sectional studies found differences in functional connectivity in the “resting state” which indicated stronger coupling between brain regions implicated in self-monitoring, cognitive control, and attention in experienced meditators compared to subjects with less or no meditation experience (Brewer et al., 2011; Jang et al., 2011; Hasenkamp and Barsalou, 2012; Taylor et al., 2012). However, little is known on the longitudinal effects of meditation training on emotional reactivity when participants are in an ordinary, non-meditative state.

In the present study, based on the extensive literature implicating the amygdala in emotion regulation, we tested the hypothesis that the amygdala response to emotional stimuli, as measured with functional magnetic resonance imaging (fMRI), would longitudinally decrease after an 8-week training in mindful-attention meditation. We also investigated how the effect might differ in another type of meditation training that has received less scientific attention so far, namely compassion meditation. The rationale for choosing these two types of meditation training is explained below.

Attention training is considered the foundation of meditation practices, as emphasized in the traditional texts (reviewed in Lutz et al., 2007; Austin, 2009). Meditation training demonstrably improves attentional skills (Valentine and Sweet, 1999; Jha et al., 2007; Tang et al., 2007; Chambers et al., 2008; Lutz et al., 2009b; MacLean et al., 2010; van den Hurk et al., 2010; reviewed in Lutz et al., 2008b; Baijal et al., 2011; Wadlinger and Isaacowitz, 2011), and theoretical accounts emphasize the role of attention regulation as one of the core components of mindfulness meditation (Brown and Ryan, 2003; Lutz et al., 2008b; Carmody, 2009; Hölzel et al., 2011b). Substantial evidence exists that attentional skills are a critical component of the emotion regulatory process (reviewed in Wadlinger and Isaacowitz, 2011), and it has been suggested that meditative interventions may be one of the most effective attention-based training methods available to improve emotional regulation (Wadlinger and Isaacowitz, 2011).

In this study, we implemented an 8-week program of mindful-attention training, in which subjects practice meditative techniques for enhancing mindful awareness of one's internal state and external environment (Wallace, 2006). This program has been used principally in the form of 3-month intensive retreats, as was the case in the Shamatha Project—a longitudinal study aimed at investigating a broad range of health-related outcomes and effects on basic physiology and brain function (MacLean et al., 2010; Jacobs et al., 2011; Sahdra et al., 2011; Saggar et al., 2012). The training includes two components of attention, which have been called focused attention (FA) and open monitoring (OM) (Lutz et al., 2008b), also known as concentrative attention and receptive attention, respectively (Brown, 1977; Valentine and Sweet, 1999; Jha et al., 2007; Austin, 2009). Three main meditative techniques are taught: mindfulness of breathing (i.e., cultivating awareness of one's breathing), mindfulness of mental events (i.e., cultivating awareness of the contents of one's mind, such as thoughts, emotions, etc.), and awareness of awareness (in which awareness itself becomes the focus of meditation).

In contrast to mindful-attention practices aimed at improving attentional skills, compassion meditation is a distinct form of contemplative practice aimed at cultivating higher levels of compassion. Compassion can be defined as the feeling that arises in witnessing another's suffering and that motivates a subsequent desire to help (Goetz et al., 2010). In the Mahayana Buddhist tradition, compassion is considered the ultimate source of well-being and happiness (Davidson and Harrington, 2001). Buddhist-inspired practices for cultivating compassion for self and others have been proposed by a number of authors as accessible methods to help alleviate psychological problems and improve well-being (Salzberg, 1995; Gilbert, 2005; Makransky, 2007; Germer, 2009; Hofmann et al., 2011; Ozawa-de Silva and Dodson-Lavelle, 2011; Jazaieri et al., 2012; Wallmark et al., 2012). Emerging scientific evidence suggests that these interventions may be beneficial on multiple levels. A pilot study indicated that compassionate mind training could lead to significant reductions in depression, anxiety, self-criticism, and shame (Gilbert and Procter, 2006). Another study suggested that compassion meditation may offer health-related benefits such as reduced immune and behavioral response to psychosocial stress (Pace et al., 2009, 2010). In a pilot study of loving-kindness meditation, a practice related to compassion meditation, chronic low back pain patients showed significant improvements in pain and psychological distress (Carson et al., 2005). Remarkably, Hutcherson et al. (2008b) found that even only a few minutes of loving-kindness meditation could increase feelings of social connection and positivity toward novel individuals. A few hours of training over the course of several days increased positive affective experiences and elicited activity in brain regions previously associated with positive affect and social affiliation (Klimecki et al., 2012). In a larger field experiment, Fredrickson et al. (2008) found that loving-kindness meditation produced increases over a 2-month period in daily experiences of positive emotions, which promoted increases in a wide range of personal resources (e.g., increased mindfulness, purpose in life, social support, decreased illness symptoms), which, in turn, predicted increased life satisfaction and reduced depressive symptoms. In a recent randomized controlled trial, an intensive meditation/emotion regulation intervention that included multiple elements of compassion training yielded reduced trait negative affect, rumination, depression, and anxiety, increased trait positive affect and mindfulness, and improved recognition of subtle facial expressions of emotion (Kemeny et al., 2012). Taken together, these recent studies support the hypothesis that compassion meditation contributes to improved emotion regulation. However, a direct comparison of mindful-attention training and compassion meditation training has been lacking.

In this study, we investigated how 8 weeks of training in either mindful-attention meditation or compassion meditation affected amygdala responses to emotional stimuli. Since we were interested in putative changes in affective trait, study participants were not instructed to enter a meditative state, so that changes in brain activity would reflect uncontrived emotional responses without being influenced by a purposeful manipulation of brain state.

## Materials and methods

### Study participants

Study participants were a subset of the subjects enrolled in a parent study being conducted at Emory University in Atlanta, GA, called the Compassion and Attention Longitudinal Meditation (CALM) study. All procedures were approved by the Institutional Review Boards at Emory University and Boston University. Healthy, medication-free adults (25–55 year-old) with no prior meditation experience were recruited in the Atlanta metropolitan area. Study participants gave written informed consent with Emory University to participate in the parent study, and additionally with Boston University to participate in the brain imaging study reported here. Participants in the parent study were randomized to eight weeks of training in either Mindful Attention Training (MAT), or Cognitively-Based Compassion Training (CBCT), or an active control intervention consisting of a health discussion group (CTRL). All interventions are described below. Fifty-one subjects (31 females, 20 males; age 34.1 ± 7.7 years, mean ± standard deviation) volunteered to participate in the brain imaging study. They underwent the pre-intervention scan before their randomization to any of the three groups. Of those who completed baseline assessments, five subjects dropped out of the study, and 10 either fell asleep or showed excessive motion in the scanner in at least one of their two scanning sessions. The final subject population in the present brain imaging study was *N* = 12 (8 females, 4 males; age 34.3 ± 9.6 years, mean ± standard deviation) in the MAT group, *N* = 12 (9 females, 3 males; age 32.0 ± 5.4 years) in the CBCT group, and *N* = 12 (5 females, 7 males; age 36.0 ± 7.6 years) in the CTRL group.

### Interventions and meditation training

All subjects participated in 2 h of class time per week for eight weeks, for a total of 16 h during the study. During these 8 weeks subjects in the MAT and CBCT groups were asked to meditate for an average of 20 min a day outside of the class. Each intervention is described in detail below. The meditation interventions were designed to be primarily experiential, with theoretical background presented only to the extent that it facilitated the meditative experience. Complete protocols for MAT, CBCT, and the active control intervention are provided in Tables 1, 2, 3, respectively.

**Table 1:** **MAT protocol**.

**Week**	**Training components**
1	**Settling the body and respiration in their natural state**
	Introduction of basic techniques for relaxing the body and settling the respiration in its natural rhythm.
2	**Mindfulness of the breathing with relaxation**
	Introduction and elaboration of practices for learning to calm the conceptually discursive mind for the purpose of attenuating involuntary thoughts. Stability of attention is practiced with the goal of sustaining attention for longer periods.
3	**Mindfulness of the breathing with relaxation and stability**
	Continuing practice of techniques designed to instill a deepening sense of physical and mental relaxation, stillness, and vigilance. When successful, involuntary thoughts subside and vividness of attention gradually increases. This gives rise to an overall sense of greater presence, calm, and equilibrium.
4	**Mindfulness of the breathing, with relaxation, stability, and vividness**
	Continuing practice of techniques designed to instill a deepening sense of physical and mental relaxation, stillness, and vigilance. When successful, involuntary thoughts subside and vividness of attention gradually increases. This gives rise to an overall sense of greater presence, calm, and equilibrium.
5	**Settling the mind in its natural state (i. e., mindfulness of mental events) (I)**
	Introduction of practices for further refining the meditator's metacognitive abilities, with the goal of attenuating the immediate and habitual absorption in one's thoughts that characterize most mental functioning. When successful, insight into the nature of the mind and its activities is achieved.
6	**Settling the mind in its natural state (II)**
	Continued practice with the goal of developing increased relaxation, stillness of awareness in the midst of mental activities, and vividness, together with heightened metacognitive abilities to observe mental states and processes without identifying with them.
7	**Awareness of awareness (I)**
	In this final technique, relaxation, stillness, and vividness of attention continue to be enhanced, leading to a perception of the process of becoming aware, as opposed to only perceiving the contents of awareness.
8	**Awareness of awareness (II)**
	As the meditator develops greater facility with this practice, the mind rests in its own luminosity and awareness. When successful, this practice leads to insight into the nature of consciousness itself.

**Table 2:** **CBCT protocol**.

**Week**	**Training components**
1	**Developing attention and stability of mind**
	Introduction of basic meditation techniques for focusing attention for increasingly longer periods of time. *These techniques are included in the practice of all subsequent compassion meditation components*.
2	**Awareness of sensations, feelings, and emotions**
	Often we are aware of only our reactions to feelings and sensations, rather than the feelings and sensations themselves. This practice hones our attention to subjective experience, and provides the meditator with practice in separating emotions and reactions.
3	**Developing compassion for oneself through the wish to emerge from suffering**
	Introduction of techniques to develop awareness of how thoughts and actions contribute to subjective experiences of happiness or suffering, and techniques to increase identification of habitual, conditioned reactions.
4	**Cultivating equanimity**
	Introducing practices designed to challenge unexamined thoughts and feelings determining categories of friend, enemy, and stranger; introducing the perspective that all persons are alike in wanting to be happy.
5	**Developing appreciation and affection**
	It is common to feel appreciation only for a few close others whose actions on our behalf are easy to observe and comprehend. Yet every day we reap the benefits of the actions of countless others. We practice becoming aware of those others, and become grateful to them.
6	**Empathy**
	Techniques will be presented for developing undifferentiated affection for others, based on the many ways that others benefit us each day. The meditators will be introduced to the concept of empathy for others: identifying with their happiness and suffering alike.
7	**Wishing and aspirational compassion**
	Using the concepts of appreciation and empathy as a starting point, the meditator will be guided toward the first stages of compassion: the wish that all beings might be happy and free of suffering, and the aspiration to help them achieve that.
8	**Active compassion for others**
	The meditation training culminates in the generation of active compassion: practices introduced to develop a determination to work actively to alleviate the suffering of others. When this training is successful, this state of mind becomes ingrained and spontaneous.

**Table 3:** **Health discussion control intervention protocol**.

**Week**	**Training components**
1	**Interacting with our environment**
	After introducing the students to each other and to the class, we will introduce the first of the top 10 things we can do to improve our health: interact with our environment, which improves mood and fosters a sense of well-being (1).
2	**The things we put in our bodies**
	The second item on the list relates to hydration, for proper physical and mental function (2). We will introduce the importance of small changes in diet for nutrition and long-term health, particularly eating breakfast (3) and eating more fruits and vegetables (4).
3	**Interacting with the healthcare system**
	This module will help participants to better understand health-related information, and to interact with healthcare providers most effectively (5).
4	**Maintaining healthy relationships**
	Strong social ties create better health, by improving immune function, protecting heart health, and warding off depression and anxiety (6).
5	**The role of exercise in physical health**
	Regular exercise reduces the risk of heart disease and helps attain and maintain a healthy weight (7).
6	**The role of exercise in emotional health**
	Regular exercise can decrease depression and anxiety and improve overall mood (8).
7	**Time out**
	Regular and sufficient sleep, as well as quiet relaxation time, are essential to physical and mental health (9).
8	**Stop stressing**
	Stress is unavoidable; the key is to recognize it. One component of stress management is learning and implementing healthier emotional expression (10).

#### Mindful attention training

In this program, subjects are trained in a set of meditation techniques for enhancing FA and mindful awareness of one's internal state and external environment. The program taught in this study was a simplified version of a comprehensive meditation training program fully described in (Wallace, 2006). The latter program is regularly taught in the form of 3-month intensive retreats, as was the case in the Shamatha Project—a longitudinal study aimed at investigating a broad range of health-related outcomes and effects on basic physiology and brain function (MacLean et al., 2010; Jacobs et al., 2011; Sahdra et al., 2011; Saggar et al., 2012). It should be noted that, while the 3-month retreat program introduces elements of compassion training as well as Buddhist ethics, these components were not taught in the MAT program in this study to avoid possible confounds with the CBCT program described below.

Each MAT class included a 50-min didactic session that introduced the meditative technique to be practiced during the week, a 30-min discussion period, and a 40-min meditation practice session. The full protocol for the MAT program is detailed in Table 1. In essence, the training includes two components of attention, which have been called FA and OM (Lutz et al., 2008b), also known as concentrative attention and receptive attention, respectively (Brown, 1977; Valentine and Sweet, 1999; Jha et al., 2007; Austin, 2009). Attention is trained by developing two complementary mental functions. One consists in attending, without forgetfulness, to the meditative object of focus (e.g., one's breathing); this faculty is called *sati* in Pali, which has been translated as awareness, bare attention, or mindfulness (Wallace, 2006). The second mental function, called introspection, is a type of metacognition that operates as the “quality control” by monitoring the meditative process and swiftly detecting the occurrence of either excitation or laxity, which are both impediments to the practice (Wallace, 1999). Three main meditative techniques are taught: mindfulness of breathing (in which the focus of attention is one's own breath), “settling the mind in its natural state,” i.e., mindfulness of mental events (in which the focus of attention is one's own mind and mental activity, such as thoughts, emotions, etc.), and awareness of awareness (in which awareness itself becomes the focus of meditation, without a specific object, so that one is simply aware of being aware). The purpose of having a meditation object that is more and more subtle or elusive as the training progresses is to increase the quality of attention. This training format closely follows standard presentations in Tibetan Buddhism and is traditionally considered appropriate for novices (Wallace, 2006). Of note, meditation practices in the MAT program bear many similarities with the practices included in the sitting meditation component of Mindfulness-Based Stress Reduction (MBSR; Kabat-Zinn, 1990). We chose to use MAT rather than MBSR because MBSR is a heterogeneous technique that involves training in mindful awareness across different practices, including not only sitting meditation but also some yoga movements and a lying-down practice called the body scan (Kabat-Zinn, 1990), which we felt would be difficult to match in the CBCT program and could potentially confound our comparison of both trainings.

#### Cognitively-based compassion training

The CBCT program was designed by Lobsang Tenzin Negi, PhD, Geshe-Lharampa, Director of the Emory-Tibet Partnership at Emory University and Spiritual Director of Drepung Loseling Monastery, Atlanta, GA. The CBCT program is based on traditional Buddhist methods for cultivating compassion (see Pace et al., 2009, 2010; Reddy et al., 2012). The CBCT program includes several meditation practices that were adapted from a set of the Mind-Training techniques (“*lo-jong*”) in the Tibetan Buddhist tradition, which derive largely from writings ascribed to the Indian Buddhist masters Śāntideva (8th century) and Atisha (11th century) (Santideva, 1997; Dalai Lama, 2001; Wallace, 2001; Jinpa, 2005). The goal of these techniques is to reverse thoughts, emotions, and behaviors that are harmful to oneself and others and to transform them into thoughts, emotions, and behaviors that are beneficial to oneself and others (Ozawa-de Silva and Dodson-Lavelle, 2011). The full protocol for the CBCT program is detailed in Table 2. The CBCT program is taught in weekly stages and includes the standard meditative practices for developing FA as preludes to deploying meditative concentration to the purposeful cultivation of specific mind states. These mind states include equanimity towards all beings, appreciation and affection for others (also known as *maitrī* in Sanskrit or *mettā* in Pali, often translated as “loving-kindness” or simply “love”), and compassion for all including oneself (*karuṇā* in Sanskrit). The training culminates with the cultivation of “active compassion”, in which meditators develop a determination to work actively to alleviate the suffering of others. The training protocol is highly iterative, and techniques introduced early in the program are practiced throughout the entire training period. As in MAT, each CBCT meditation class includes a 50-min didactic session that describes the meditative technique introduced during the week, a 30-min discussion period, and a 40-min meditation practice session.

In both meditation interventions, participants received detailed instructions pertaining to the meditative technique that they were to practice in class and then at home for the following week. Our meditation instructors employed various pedagogical methods for making these practices accessible, for example using various metaphors, real-life examples, and stories. A considerable amount of time was spent explaining the potential applications of these practices in everyday life. A fair portion of the discussion periods was also spent reviewing the meditation practice to make sure that the participants had a grasp of the practice, skills, and concepts at hand.

#### Health discussion control intervention

This active control intervention was adapted from a university-level health education class designed by Daniel D. Adame, MSPH, PhD, CHES, Associate Professor of Health Education at Emory University (retired). This course (PE101) was mandatory for all Emory University freshmen for many years until Dr. Adame's retirement. The intervention used in the present study was designed and taught by two MPH students of Dr. Adame's based on material from discussion sections that were key components of the PE101 course. Both teachers were fully convinced of the utility of this health education intervention, which was deemed worthy of university credit at Emory University. The active control group met for 2 h per week for 8 weeks, exactly matching the class time commitment required for both MAT and CBCT training. Each discussion class focused on a topic of direct relevance to emotional and/or physical health in adults (see Table 3). After a didactic talk on the topic of the week, subjects actively participated in small group discussions, role-playing scenarios, and friendly debates designed to make the health topics covered personally relevant. At the start of the first class of each week, subjects completed a brief questionnaire querying which aspects of the health topic from the previous week (if any) they had started implementing in their daily lives.

### Self-report assessments

As part of the CALM study, participants filled the Beck Depression Inventory (BDI-II) and the Beck Anxiety Inventory (BAI) before and after the 8-week intervention. In addition, those in the CBCT and MAT groups were asked to log on a daily basis the amount of time that they spent practicing meditation at home.

### Brain imaging (fMRI) experiment

Volunteers for the brain imaging study took part in two scanning sessions, one within the 3 weeks preceding the intervention (PRE) and one within 3 weeks after the intervention (POST). All scanning took place at the Athinoula A. Martinos Center for Biomedical Imaging, a joint center of the Harvard-MIT Division of Health Sciences and Technology and the Massachusetts General Hospital Radiology Department. The MRI scanner was a Siemens 3T Tim-Trio scanner with vendor-supplied 32-channel head array coil. Each scanning session included a high-resolution (1-mm^3^ voxel) T_1_-weighted anatomical scan using multi-echo magnetization-prepared rapid gradient-echo (MEMPRAGE) imaging (van der Kouwe et al., 2008), and a 35-min T^*^_2_-weighted blood-oxygenation level-dependent (BOLD) fMRI experiment. Functional images (108 volumes per functional run) were obtained with gradient-echo Echo-Planar Imaging (EPI) using the following parameter values: TE = 30 ms, TR = 3 s, voxel size = 3 × 3 × 3 mm, bandwidth = 2240 Hz/px, matrix size 72 × 72, and 47 slices with no gap.

During the fMRI experiment participants were presented photographs from the IAPS database (Lang et al., 2005). All selected images depicted people in various settings, with equally distributed positive, negative, or neutral emotional valences (with 72 images in each category for a total of 216 images). The instructions given to the subjects were as follows: “Please press the button every time you see a picture appear on the screen. You'll notice that these images have various emotional contents. Just watch the images and let yourself react to them naturally.” The simple button press task, without rating or labeling the image, was chosen to keep participants engaged while minimizing cognitive load, which is known to interfere with neural activation in emotion-associated brain regions (Critchley et al., 2000; Hariri et al., 2000; Liberzon et al., 2000; Taylor et al., 2003). The order of the images was randomized across sessions and across subjects, such that each subject viewed all 216 images once over the two scanning sessions (PRE and POST), but in a different order. Each session consisted of six runs; during each run 18 images were presented, for a total of 108 images during the whole session. Each image was presented for 5 s followed by a 13 s blank (gray) screen of similar overall luminance. This mixed block/event-related design was chosen to allow the BOLD signal to return to baseline between images, which increases functional signal-to-noise ratio, statistical power, and robustness of the results (Amaro and Barker, 2006). The duration of each block was short enough to prevent habituation in the amygdala (Haas et al., 2009).

To prevent unintentional influences on the study participants, which may have confounded our results, the experimenters (GD and ELS) were blinded with respect to subjects' group assignment until after the end of the post-intervention fMRI experiment.

We attempted to assure that the participants did not enter a meditative state during the fMRI experiment. During the pre-intervention scan, all subjects were meditation-naïve, and therefore did not have any training that would allow them to enter such a state. During the post-intervention scan, the use of the word “meditation” was carefully avoided by the experimenters during all interactions, and the participants were never primed about their meditation training (or lack thereof in the case of subjects in the control group). During the debriefing at the end of the scan, we confirmed that the subjects did not enter a meditative state by asking them: “Were you meditating during the image presentation?” to which all of them replied in the negative. Some subjects then asked, “Was I supposed to meditate?” to which we replied that they were not supposed to meditate, and that in fact we expected them not to meditate.

### Data analysis

**Figure 1:**
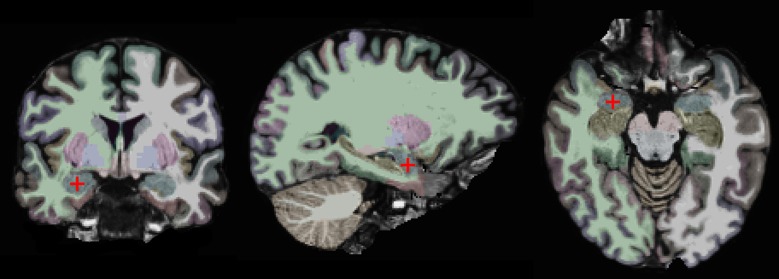
**Coronal, sagittal, and horizontal views of the brain of one study participant.** The right amygdala is marked by a red crosshair and colored in blue. The other colors indicate different brain regions as automatically segmented by the FreeSurfer software.

Our approach is based on a region-of-interest (ROI) analysis (Nieto-Castanon et al., 2003; Poldrack, 2007). Two anatomically defined ROIs, the left and right amygdalae, were automatically segmented in each subject's individual anatomical brain scan with the FreeSurfer (v. 5.1) image analysis suite, which is documented and freely available for download online (http://surfer.nmr.mgh.harvard.edu/) (Fischl et al., 2002, 2004). This segmentation is shown on one of our subjects in Figure 1. One advantage of using anatomically defined ROIs is that it eliminates the potential problem of circular analysis that exists for functionally defined ROIs (Poldrack, 2007; Kriegeskorte et al., 2009; Poldrack and Mumford, 2009; Vul et al., 2009). There are other advantages of using an ROI-based approach. First, it removes some variability due to noise by averaging over all voxels in the ROI. Second, it is more statistically powerful than other methods, since it controls for Type I errors by limiting the number of statistical tests. Third, it enables precise spatial correspondence of the region-of-interest across subjects, since it does not involve normalizing different brains to a common atlas (Poldrack, 2007).

The fMRI data were analyzed using FSL (www.fmrib.ox.ac.uk/fsl) (Smith et al., 2004; Woolrich et al., 2009) and Matlab 7.11 (The MathWorks, Inc., Natick, MA). Preprocessing included brain extraction, slice-timing correction, motion correction, B_0_ unwarping, temporal high-pass filtering, and registration to anatomical scan for each individual subject. The first 9 s of each run (before the first image presentation) were discarded to eliminate any transverse magnetization equilibration effects. For each run, the three categories of pictures (negative, neutral, and positive) were included as separate explanatory variables in the model. In addition, the global BOLD signal intensity (averaged across all within-brain voxels) was included as a nuisance variable. ROI analysis was performed in each subject's native space by averaging contrasts of parameter estimate (COPE) values in the left and right amygdala separately. These contrast maps were used as input for further statistical analyses in Matlab. In addition, for data exploration purposes a whole-brain statistical parametric mapping analysis was also conducted in FSL.

## Results

### Amount of meditation practice

The total reported duration of meditation practice in the MAT group was 645 ± 340 min (mean ± standard deviation, *N* = 12), ranging from 210 to 1491 min. In the CBCT group it was 454 ± 205 min (*N* = 12), ranging from 190 to 905 min. There was no significant difference between the two groups (two-sample *t*-test, *p* = 0.27, *t* = −1.13).

### BAI and BDI

Before the interventions, the BAI scores of all participants who completed the Boston study and the questionnaires were 2.6 ± 3.1 in the MAT group (*N* = 13), 4.6 ± 4.1 in the CBCT group (*N* = 17), and 4.0 ± 4.2 in the control group (*N* = 11), and BDI scores were 2.8 ± 3.8 in the MAT group, 5.8 ± 7.9 in the CBCT group, and 3.4 ± 4.4 in the control group. After the interventions, BAI scores were 1.8 ± 2.5 in the MAT group, 3.8 ± 4.2 in the CBCT group, and 1.3 ± 2.0 in the control group, and BDI scores were 2.8 ± 5.2 in the MAT group, 2.7 ± 4.1 in the CBCT group, and 2.4 ± 3.1 in the CTRL group. A single-factor repeated-measure ANOVA revealed a significant effect of time on BAI scores [ANOVA *F*_(1, 38)_ = 13.23, *p* = 0.0008] and on BDI scores [ANOVA *F*_(1, 38)_ = 6.33, *p* = 0.016], but no effect of group. The group × time interaction was not statistically significant for BAI scores [*F*_(2, 38)_ = 2.41, *p* = 0.10], nor for BDI scores [*F*_(2, 38)_ = 2.42, *p* = 0.10]. Nonetheless, an exploratory analysis suggested that BDI scores were significantly reduced from pre- to post-training in the CBCT group (mean difference −3.1 ± 4.7, two-tailed paired *t*-test, *p* = 0.015, *t* = −2.72, *df* = 16), but not in the other groups.

In the two meditation groups, the amount of time that participants reported practicing meditation was not correlated with pre- or post-intervention BDI or BAI scores, nor with their PRE-POST differences (*r*^2^ < 0.1, *p* > 0.2 in all cases).

### Brain activation in the amygdala

**Figure 2:**
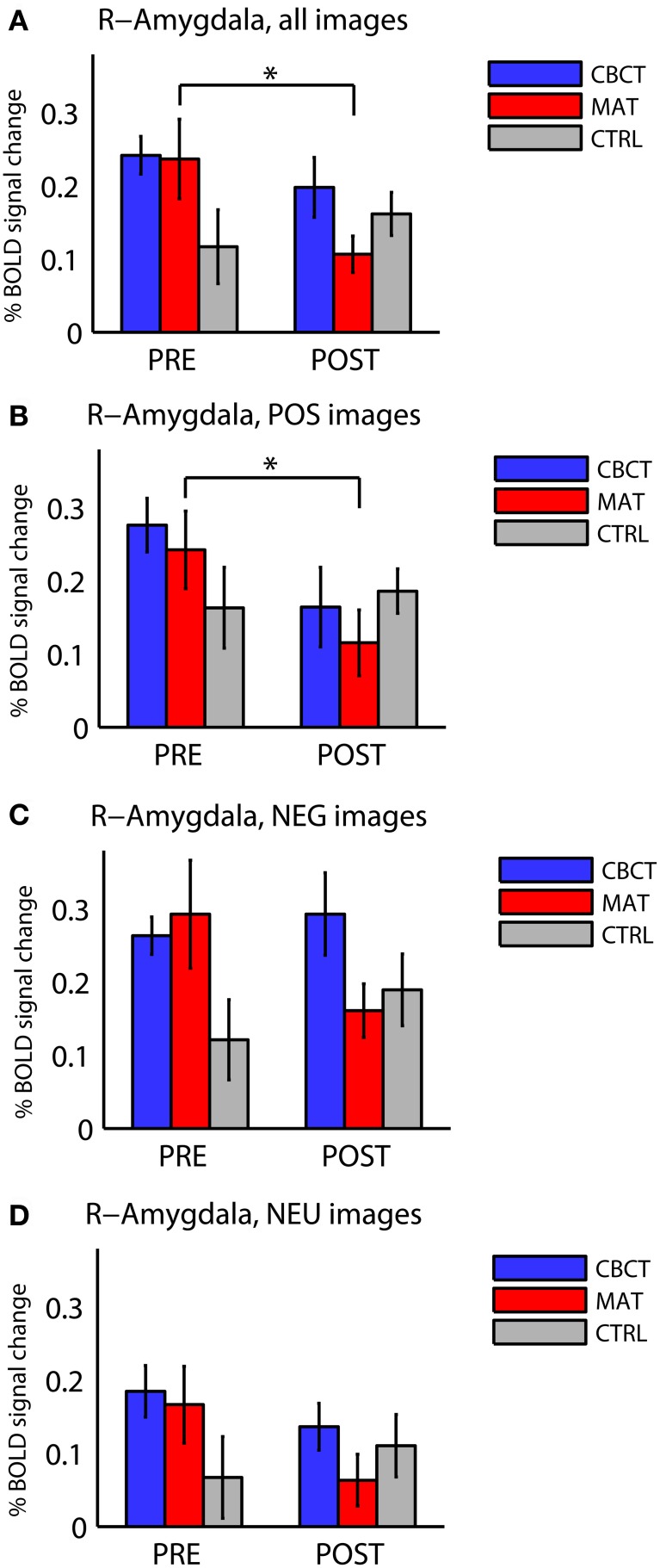
**Percentage BOLD signal change in right amygdala for all three groups of subjects (CBCT, MAT, CTRL), in the pre-intervention scan (PRE) and in the post-intervention scan (POST), (A) for images of all valences, (B) for images with positive valence (POS), (C) for images with negative valence (NEG), and (D) for images with neutral valence (NEU).** The asterisks indicate statistically significant differences between PRE and POST (two-tailed paired *t*-tests, *p* < 0.05). Bars represent mean ± standard error.

The different activation levels in the right amygdala, across image valences and across groups, are summarized in Figure 2. No effects or trends were found in the left amygdala.

The three groups did not differ significantly from each other before the intervention. Although Figure 2A seems to show a difference in right amygdala activation across the three groups before the training (PRE)—with the CTRL group seemingly showing lower activation than the other two groups—this difference did not reach statistical significance [one-way ANOVA: *F*_(2, 33)_ = 2.22, *p* = 0.12, *df* = 35]. This trend may be explained by the higher proportion of male subjects in the CTRL group. Indeed, regrouping all subjects by gender in PRE revealed a statistically significant group difference with respect to gender, with females showing higher activation in the right amygdala in response to all images (0.24 ± 0.19, mean ± standard deviation in units of percentage change in BOLD signal, *N* = 22) compared to males (0.13 ± 0.11, *N* = 14; group difference: two-sample *t*-test, *p* = 0.038, *t* = 2.17).

When comparing right amygdala activation before the intervention (PRE) and after the intervention (POST), we found a significant Group × Time interaction in response to images of all valences [repeated-measure ANOVA *F*_(2, 33)_ = 3.73, *p* < 0.035]. The PRE-POST difference was significantly greater in MAT than in CTRL, as revealed by Tukey's Honestly Significant Difference test for multiple comparisons [estimated difference between MAT and CTRL: −17.5, confidence interval (−33.2, −1.8)], whereas the CBCT group did not differ significantly from the other two groups.

In within-group analyses, no significant effects or non-significant trends were observed in the CTRL group (who did not practice meditation). In the MAT group, we found a longitudinal (PRE to POST) decrease in right amygdala activation in response to images of all valences overall (two-tailed paired *t-test, p* = 0.012, *t* = −3.00, *df* = 11, Figure 2A), and in response to positive-valence images (two-tailed paired *t*-test, *p* = 0.011, *t* = −3.06, *df* = 11, Figure 2B). While the response to negative-valence images decreased as well, this trend was not statistically significant (two-tailed paired *t*-test, *p* > 0.1, Figure 2C). The response to neutral-valence images did not vary (two-tailed paired *t*-test, *p* > 0.1, Figure 2D).

**Figure 3:**
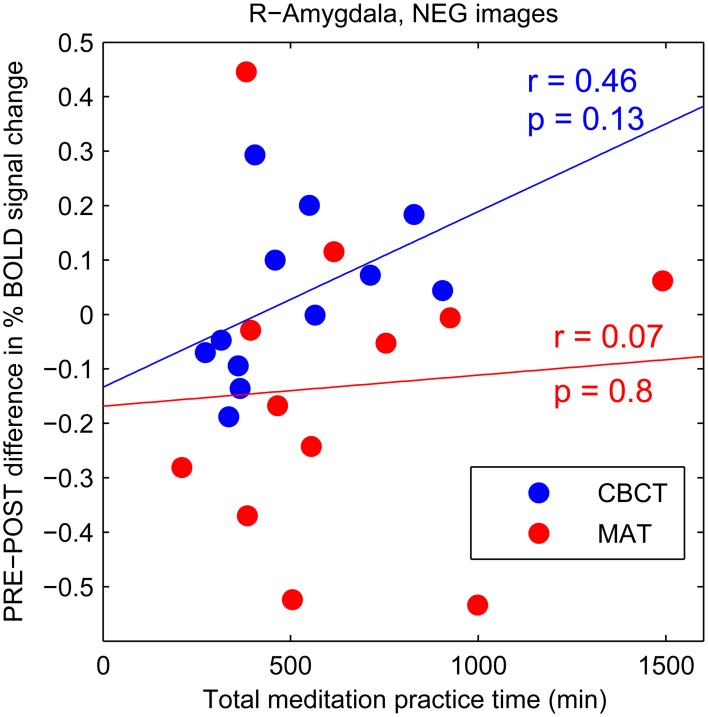
**PRE-POST difference in percentage BOLD signal change in right amygdala as a function of total meditation practice time.** Each data point corresponds to an individual subject. The CBCT group is shown in blue, the MAT group in red. Linear regression lines are shown in corresponding colors.

The CBCT group also exhibited a decrease in right amygdala activation in response to positive-valence images, by a similar amount as the MAT group on average (mean: −0.112 in CBCT, −0.127 in MAT), but it did not reach statistical significance (two-tailed paired *t*-test, *p* = 0.085, *t* = −1.89, *df* = 11, Figure 2B). The response to neutral-valence images did not vary (two-tailed paired *t*-test, *p* > 0.1, Figure 2D). Interestingly, we found a trend increase in right amygdala activation in response to negative-valence images. While this increase was not significant at the group level (two-tailed paired *t*-test, *p* > 0.1, Figure 2C), a correlation analysis with the amount of meditation practice time indicated that increased amygdala activation occurred in the subjects who had reported the most hours of practice, while those who had practiced less showed a small decrease in amygdala activation. However, this correlation between practice time and PRE-POST difference in amygdala activation did not reach statistical significance (correlation coefficient *r* = 0.46, *p* = 0.13, *N* = 12, Figure 3). For completeness, we performed the same analysis in the MAT group but found no effect of practice time (correlation coefficient *r* = 0.07, *p* = 0.8, Figure 3). In both groups, there was no evidence of an effect of practice time on the PRE-POST difference in amygdala response in the case of positive or neutral images.

**Figure 4:**
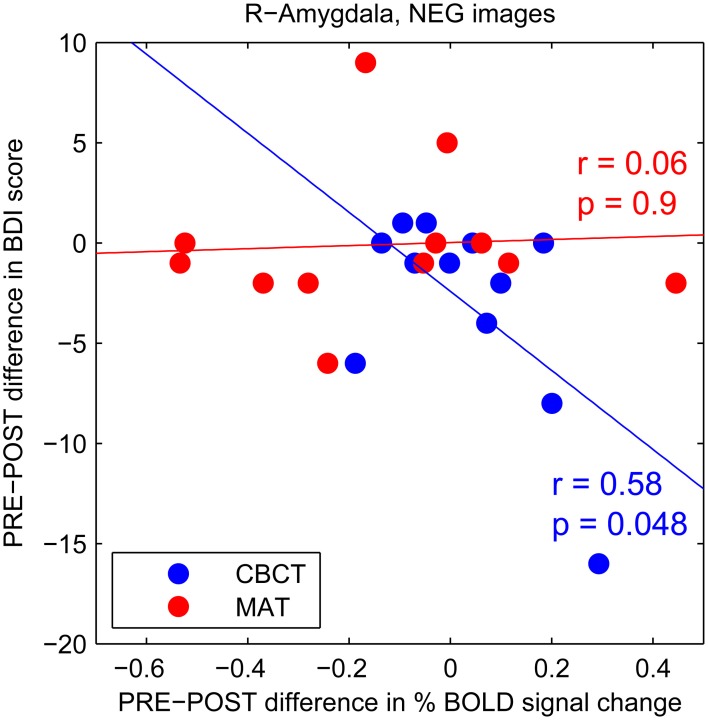
**PRE-POST difference in depression score as a function of PRE-POST difference in percentage BOLD signal change in right amygdala.** Each data point corresponds to an individual subject. The CBCT group is shown in blue, the MAT group in red. Linear regression lines are shown in corresponding colors.

To further investigate this trend increase in amygdala activation in response to negative images after CBCT training, we performed a correlation analysis between the PRE-POST difference in amygdala activation and the PRE-POST difference in depression (BDI) scores. We found a statistically significant negative correlation in the CBCT group (*r* = 0.58, *p* = 0.048, *N* = 12, Figure 4). In other words, a greater increase in amygdala activation in response to negative images was associated with a greater decrease in depression score after CBCT training. No such correlation was found after MAT training (*r* = 0.06, *p* = 0.9, *N* = 12, Figure 4). No correlation was found in the case of images of positive of neutral valence in either the MAT or CBCT group (*p* > 0.5 in all cases). Neither MAT nor CBCT group showed correlations between the difference in amygdala activation and the difference in anxiety (BAI) scores (*p* > 0.3 in all cases).

### Whole-brain analysis

For data exploration purposes, a whole-brain statistical parametric mapping analysis was also conducted. It did not reveal any significant PRE-POST differences at the whole-brain level in any of the three groups.

## Discussion

The longitudinal effects of meditation training in beginners, in terms of brain function, are only beginning to be elucidated. Based on the literature showing that the amygdala plays a prominent role in emotional processing and attention, and that amygdala activation in response to emotional stimuli varies with personality trait and with different meditative states, we hypothesized that amygdala response to emotional stimuli when subjects were in a non-meditative state would decrease longitudinally after 8 weeks of training in mindful-attention meditation. We found a longitudinal decrease in right amygdala activation in response to positive images, and in response to images of all valences overall, after training in mindful-attention meditation. No difference or trends were found in the control group between the pre- and post-intervention scans. Because of the longitudinal, controlled design of our study, our results support our hypothesis that participation in an 8-week mindful-attention meditation training would cause a reduction in amygdala response to emotional stimuli while participants were not meditating. However, the case of compassion meditation training was less straightforward, as we discuss below.

Previous studies have implicated the amygdala in the effects of meditation training, both in beginner and expert meditators. In beginner meditators after only 1 week of practice, Taylor et al. (2011) reported a down-regulation of the amygdala during viewing emotional images when the subjects were instructed to enter a “mindful” meditative state, compared to a baseline, non-meditative state. In a longitudinal study of MBSR for patients with social anxiety disorder, Goldin and Gross (2010) reported that after MBSR training, patients exhibited a faster return to baseline in their right amygdala activation while viewing phrases of negative self-beliefs, which in these patients can be considered a form of emotional stimulus with negative-valence. These subjects also showed decreased negative emotion ratings and increased activity in brain regions implicated in attentional deployment (Goldin and Gross, 2010). These two studies point to a decrease in amygdala activation after mindfulness meditation training in beginners.

Studies involving individuals with extensive meditation practice depict a more complex picture. In one study, experienced meditators showed lower amygdala activation in response to emotional distracters when in a meditative state of mindful-attention (“one-pointed concentration”) compared to a non-meditative, baseline state (Brefczynski-Lewis et al., 2007). The authors also found a negative correlation between the number of hours of meditation training and right amygdala activation during concentration meditation in a group of experienced meditators while hearing negative-valence emotional sounds, which might indicate that more experience with meditation leads to improved ability to down-regulate the amygdala. However, another study found no effect in the amygdala when experienced meditators were instructed to pay mindful-attention to emotional images compared to a non-meditative, baseline state (Taylor et al., 2011). A possible explanation for the discrepancy between these two studies is that subjects may have deployed attention differently. In the Brefczynski-Lewis et al. (2007) study, the emotional sounds were distracters, a condition which may have required some down-regulation. In the Taylor et al. (2011) study, however, the subjects were instructed to take the emotional pictures as the focus of their mindful-attention meditation. Commenting on the apparent lack of amygdala down-regulation that they found in experienced meditators when comparing the Mindful condition with the Baseline (non-meditative) condition, Taylor et al. suggested that the long-term practice of meditation may lead to emotional stability by promoting acceptance of emotional states and enhanced present-moment awareness, rather than by eliciting control over low-level affective cerebral systems from higher-order cortical brain regions. We propose that the lack of change in amygdala activation across conditions (Mindful vs. Baseline) that Taylor et al. found in experienced meditators may also indicate that the Baseline state in these subjects is more similar to the Mindful state. Indeed, the only differences between Mindful and Baseline states reported by Taylor et al. in experienced meditators consisted in deactivations in regions of the default-mode network, with no difference in brain areas associated with emotional processing. In this light, the results from Taylor et al. may be an indication that the Baseline state in experienced meditators is rather similar to their Mindful state and differs from the Baseline state in non-meditators, consistent with other neuroimaging studies which found differences in resting state (Brewer et al., 2011; Jang et al., 2011; Hasenkamp and Barsalou, 2012; Taylor et al., 2012) and in brain morphometry (Lazar et al., 2005; Pagnoni and Cekic, 2007; Grant et al., 2010; Luders et al., 2011, 2009) in meditators versus non-meditators.

Our results reported here are also consistent with the hypothesis that meditators display differences with non-meditators in terms of brain function, in particular in how the amygdala is activated in response to a passive emotional challenge. In addition, our results demonstrate that changes in brain function, in a non-meditative state, after 8 weeks of meditation training can be measured longitudinally in a non-clinical population. These results support the more general hypothesis that meditation training can promote enduring changes in mental function, i.e., in the development of certain traits (Lutz et al., 2007, 2008b; Slagter et al., 2011).

### Putative mechanisms

Emotion regulation relies on attentional capabilities (Gross, 1998; Ochsner and Gross, 2005, 2008), and it has been proposed that emotion regulation can be improved by attention training, especially in the context of meditation training (Wadlinger and Isaacowitz, 2011). Given that the amygdala plays an essential role in both attention and emotion regulation, it is possible that our finding of a reduced amygdala response to emotional stimuli after mindful-attention training can be explained by an improvement in attentional skills. Indeed, a growing number of studies supports the view that meditation training improves attentional skills (Valentine and Sweet, 1999; Jha et al., 2007; Tang et al., 2007; Chambers et al., 2008; Lutz et al., 2009b; MacLean et al., 2010; van den Hurk et al., 2010; Baijal et al., 2011; reviewed in Lutz et al., 2008b; Wadlinger and Isaacowitz, 2011), and theoretical accounts emphasize the role of attention regulation as one of the core components of mindfulness meditation (Brown and Ryan, 2003; Lutz et al., 2008b; Carmody, 2009; Hölzel et al., 2011b).

It was difficult to experimentally assess the level of attention that subjects deployed in our task, because we did not want to include an attention-demanding cognitive task which may have confounded the emotional response as it would normally occur outside the laboratory. Indeed, it is well-established that an experimental task consisting in rating or labeling emotions reduces activation in the amygdala compared to passive viewing or to a match-to-sample task (Hariri et al., 2000; Taylor et al., 2003; Hutcherson et al., 2008a), which has led to the suggestion that attention alters the salience of some aspects of the emotional events with which the amygdala is concerned (Hutcherson et al., 2008a). Nevertheless, our results are consistent with the possibility that MAT participants dedicated more attentional resources to the images after meditation training than before training.

Another possibility is that mindful-attention training enhanced participants' baseline positive affect, which would make the effect of positive-valence stimuli on the amygdala comparatively smaller. This would be consistent with previous findings that mindfulness-based interventions were associated with lowered intensity and frequency of negative affect (Brown and Ryan, 2003; Chambers et al., 2008), and that heightened states of mindfulness were associated with both higher positive affect and lower negative affect (Brown and Ryan, 2003). In addition, dispositional mindfulness has been associated with less alarming stress appraisals, more approach-oriented coping, and less avoidant coping (Heppner, 2007; Weinstein et al., 2009). If indeed our study participants showed higher positive affect after mindful-attention training (which we did not measure directly), this would also be consistent with our finding that amygdala reactivity was overall lower in response to images of all emotional valences.

### Case of compassion meditation

One aspect of our study consisted in exploring the effects of compassion meditation training on amygdala activation. As cited above, compassion can be defined as the feeling that arises in witnessing another's suffering and that motivates a subsequent desire to help (Goetz et al., 2010) [for similar definitions, see Halifax (2012); Lazarus (1991); Nussbaum (1996, 2001)]. In this view, compassion is an affective state defined by a specific subjective feeling which is related to empathy or empathic concern (reviewed in Goetz et al., 2010). Compassion requires the ability to understand the feelings or emotional states of others, which includes two major components: affective empathy, which is the ability to experientially (i.e., emotionally, “viscerally”) share the affective states of others; and cognitive empathy, or the ability to take the mental perspective of others and make inferences about their mental or emotional states (Shamay-Tsoory, 2011; Cox et al., 2012). Both components were included in the compassion meditation training that we used in this study.

There exist important differences between compassion meditation and mindful-attention meditation as meditative states. For example, in contrast to the previous studies discussed above, experienced meditators showed *higher* amygdala activation in response to emotionally charged sounds of human vocalizations (i.e., a baby cooing, a woman screaming) when in a meditative state of non-referential compassion, compared to when they were in a non-meditative, baseline state (Lutz et al., 2008a). The meditative state of non-referential compassion was also accompanied by an increase in heart rate which was significantly associated with brain activation in several brain areas involved with the modulation of bodily arousal states, suggesting that the compassion meditative state was one of higher arousal (Lutz et al., 2009a).

Currently, the role of the amygdala in empathy and compassion is still not clear. While the amygdala is usually not considered part of the core brain network for empathy (Fan et al., 2011; Shamay-Tsoory, 2011), several neuroimaging studies of empathy and compassion have implicated the amygdala. Increased amygdala activation was reported in several empathy-related tasks, especially in females (Derntl et al., 2010; Klimecki et al., 2012), and was also reported in response to hearing people or infants crying, stimuli that presumably elicit compassion (Sander and Scheich, 2001, 2005; Sander et al., 2007). Some evidence suggests that bilateral amygdala damage disrupts affective empathy, but not cognitive empathy, indicating that the amygdala may play an important role in affective empathy (Hurlemann et al., 2010). In a recent study, dominance of affective empathy compared to cognitive empathy was associated with stronger functional connectivity among social-emotional brain regions which included the amygdala (Cox et al., 2012).

Together, these previous studies suggest that compassion (as an affective state) is associated with higher amygdala activation. Our finding of a trend increase in amygdala activation after compassion meditation training in response to negative-valence images is consistent with those previous results—especially in light of the fact that all photographs that we used as visual stimuli depicted human beings, such that the negative-valence images typically showed people in various situations of suffering. One might speculate that these images of suffering inspired more compassion in the participants after compassion training, which may itself be related to an increase in amygdala activation, as seen in experts (Lutz et al., 2008a). We propose two reasons why this increase in amygdala response did not reach statistical significance at the group level in our study. First, unlike in previous studies, in our task the subjects were not instructed to specifically cultivate compassion or empathy, nor to enter a meditative state. While this experimental condition was chosen because it is more relevant to everyday life and could reveal changes in trait rather than mere changes in brain states, it has the disadvantage of yielding a smaller effect size. This raises the possibility that our study simply did not have sufficient statistical power, with our relatively small sample size of twelve subjects per group. Future studies with larger cohorts are needed to address this possibility. Second, the training in compassion meditation also included a substantial amount of practice in mindful-attention meditation, which is considered foundational to meditation in general (see Table 2). Therefore, we might expect the subjects in the compassion meditation group to show some of the same effects from this training as the subjects in the MAT group. If indeed mindful-attention meditation has the effect of reducing amygdala response to negative stimuli while compassion meditation has the effect of enhancing it (as suggested by the previous studies mentioned above), these two effects may counteract each other in subjects who practiced both types of meditation, i.e., in the CBCT group. Interestingly, the CBCT participants who reported the least amount of meditation practice showed a trend decrease in the amygdala response to images of human suffering, similar to the trend decrease found in the MAT group (see Figure 3). This raises the possibility that the CBCT participants who practiced less showed only the effect of the mindful-attention aspect of the training, and that the specific effects of compassion training only appeared in the subjects who practiced more.

In the case of compassion meditation training, we also found that a greater increase in amygdala response to images of suffering was associated with a greater decrease in depression score (see Figure 4). This finding is especially intriguing as it seems to contradict the well-known association between clinical depression and enhanced amygdala response to negative-valence stimuli (reviewed in Drevets et al., 2008). However, these previous studies typically used non-contextual images of sad or angry faces; such stimuli may be less likely to elicit compassion than the more situated images from the IAPS database used in the present study. While only few studies to date have directly investigated compassion or empathy in conjunction with depression, they seem to suggest that acute depression is associated with impaired empathic abilities and that empathy improves with remission (Donges et al., 2005; Cusi et al., 2011). If so, our finding of a greater increase in amygdala response associated with a greater decrease in depression score may be explained by an increased capacity for compassion after compassion training.

### Possible gender effects

Although our study was not designed to investigate the effects of gender on meditation training (our sample being too small in size and not balanced across gender), we found a trend which was almost statistically significant (*p* = 0.059) suggesting a gender difference in the amygdala response to all images at baseline (i.e., in the pre-intervention scan), with females showing higher activation in the right amygdala in response to emotional images compared to males. This is consistent with previous studies that reported a significant interaction of emotional valence and gender of participants on brain activation, particularly affecting the amygdala (Killgore and Yurgelun-Todd, 2001; Wager et al., 2003; Wrase, 2003; Proverbio et al., 2009). A recent study also found that females showed stronger brain activation in three different empathy tasks (emotion recognition, perspective taking, and affective responsiveness) in several emotion-related areas, including the amygdala (Derntl et al., 2010). More generally, some studies reported differences across gender in the engagement of emotion regulation strategies (Thayer et al., 2003; Matud, 2004). Overall, these gender differences raise the possibility that gender may act as a moderator on the longitudinal effects of meditation training on amygdala activation. Future studies will be needed to directly investigate this question.

## Concluding remarks

In this study, 8 weeks of training in two different forms of meditation yielded distinct changes in amygdala activation in response to emotionally valenced images while the subjects were in an ordinary, non-meditative state. This finding suggests that meditation training may affect emotional processing in everyday life, and not just during meditation. This is consistent with the hypothesis that the cultivation of specific meditative states, which are relatively short-term, can result in enduring changes in mental function, i.e., in the long-term development of certain traits (Slagter et al., 2011). Future research is needed to investigate the longitudinal impact of meditation training on other brain areas involved with affective response, emotion regulation, and attention.

### Conflict of interest statement

The authors declare that the research was conducted in the absence of any commercial or financial relationships that could be construed as a potential conflict of interest. In the interest of full disclosure, we would like to report that, in the previous 12 months, CLR has consulted for Bristol Myers Squibb and Pamlab, and has prepared and presented disease-state promotional material for Pamlab.
